# Management of pulmonary aspergillosis in children: a systematic review

**DOI:** 10.1186/s13052-023-01440-9

**Published:** 2023-03-28

**Authors:** Vito Terlizzi, Marco Antonio Motisi, Roberta Pellegrino, Luisa Galli, Giovanni Taccetti, Elena Chiappini

**Affiliations:** 1 Cystic Fibrosis Regional Reference Center, Department of Paediatric Medicine, Meyer Children’s Hospital IRCCS, Florence, Italy; 2 Pediatrics resident, Department of Health Sciences, Meyer Children’s University Hospital IRCCS, University of Florence, Florence, Italy; 3 Infectious Diseases Unit, Department of Health Sciences, Meyer Children’s University Hospital IRCCS, University of Florence, Florence, Italy

**Keywords:** PULMONARY aspergillosis, Children, Galactomannan, β-D-glucan, Voriconazole

## Abstract

Invasive pulmonary aspergillosis (IPA) is a severe condition in immunocompromised children, but the optimal management is still under debate. In order to better clarify this issue, a literature search was performed through MEDLINE/PubMed database to describe current risk factors and diagnostic, therapeutic and prophylactic tools for invasive pulmonary aspergillosis (IPA) in the paediatric age. Observational studies and clinical trials regarding diagnosis, treatment and prophylaxis were considered, and results were summarised. Five clinical trials and 25 observational studies (4453 patients) were included.

Haematological malignancies, previous organ transplant and other primary or acquired immunodeficiency were identified as risk factors for IPA in children.

Current diagnostic criteria distinguish between "proven", "probable" and "possible" disease. Consecutive galactomannan assays have good sensitivity and specificity, especially when performed on broncho-alveolar lavage. At the same time, β-D-glucan should not be used since cut-off in children is unclear. PCR assays cannot currently be recommended for routine use.

Voriconazole is the recommended first-line agent for IPA in children older than 2 years of age. Liposomal amphotericin B is preferred in younger patients or cases of intolerance to voriconazole. Its plasma concentrations should be monitored throughout the treatment. The optimal duration of therapy has yet to be determined. Posaconazole is the preferred prophylactic agent in children older than 13 years old, whereas oral voriconazole or itraconazole are the drugs of choice for those between 2–12 years. Further good-quality studies are warranted to improve clinical practice.

## Main text

### Introduction

*Aspergillus* spp. is a ubiquitous, slow-growing mold that commonly colonises the respiratory tract. Depending on the host immune status and lung structure, it can manifest as different clinical entities, such as aspergilloma, allergic bronchopulmonary aspergillosis (ABPA), allergic sinusitis, invasive aspergillosis, chronic pulmonary aspergillosis [1]. Allergic sinusitis and ABPA are allergic responses to colonisation by *Aspergillus* spp. occurring in immunocompetent patients. In particular, ABPA affects children with asthma, causing frequent flare-ups, and/or cystic fibrosis (CF) [2]. Invasive pulmonary aspergillosis occurs in immunocompromised children with impaired neutrophil and T-lymphocyte function and children with chronic pulmonary diseases or CF. This severe disease requires prompt treatment, but the diagnosis and management of IPA in children are still challenging and often delayed. Environmental and medical prophylaxis is essential in patients with risk factors, and in such cases, clinical and radiological suspicion is sufficient to initiate empirical therapy with antifungal agents. Since most of the available studies concerning IPA are carried out in adults, we performed a systematic literature review with the aim of summarising the currently available data regarding IPA in pediatric age with a focus on diagnostic tools, treatment and prophylaxis.

## Methods

The authors identified the following five key questions:

1. What are the main risk factors for IPA in children?
2. What are the main diagnostic tools for IPA in children?
3. What is the first-line agent for IPA in children?
4. What is the optimal duration of treatment for IPA in children?
5. What are the main prophylactic drugs used for IPA in children?

A systematic review of the literature was performed in line with the Preferred Reporting Items for Systematic Reviews and Meta-analyses (PRISMA) guideline recommendations [3]. The research was conducted through MEDLINE/PubMed, including articles published from the 1st of January 2002 to the 21st of December 2021. References of all relevant articles were also evaluated, and pertinent articles were included. The search strings were as follows: “(Invasive[Title/Abstract]) AND (Aspergillosis[Title/Abstract]) AND (Children[Title/Abstract] OR Paediatric[Title/Abstract])” and “(Invasive[Title/Abstract]) AND (Aspergillosis[Title/Abstract]) AND (Pulmonary[Title/Abstract] OR Lung [Title/Abstract]) AND (Children[Title/Abstract] OR Paediatric[Title/Abstract])”.

### Inclusion and exclusion criteria

The research was restricted to English language. Articles reporting risk factors, diagnostic tools, treatment and prophylaxis for IPA in paediatric population (age < 18 years) with a sample size greater than ten were included independently from the study design. Review articles, case reports, commentaries, editorials, letters to the author and pre-print records were excluded as well as studies referring to adult populations.

### Data extraction

Duplicate publications were removed, then two authors separately (RP and MAM) checked the titles and abstracts and removed irrelevant studies according to the inclusion and exclusion criteria. Pertinent articles from the bibliographic references of the selected studies were also considered, and an additional review of the literature was performed prior to final drafting. Articles were categorized according to the study design. Data about IPA risk factors, diagnostic tools, treatment, and prophylaxis were extracted.

### Quality assessment

The Jadad scale was used to assess quality for RCTs [4], while the Methodological Index for Non-randomized Studies (MINORS) was used for non-randomized ones [5]. Observational studies were evaluated for adherence to Strengthening the Reporting of Observational Studies in Epidemiology (STROBE) recommendations [6].

## Results

**Fig. 1:**
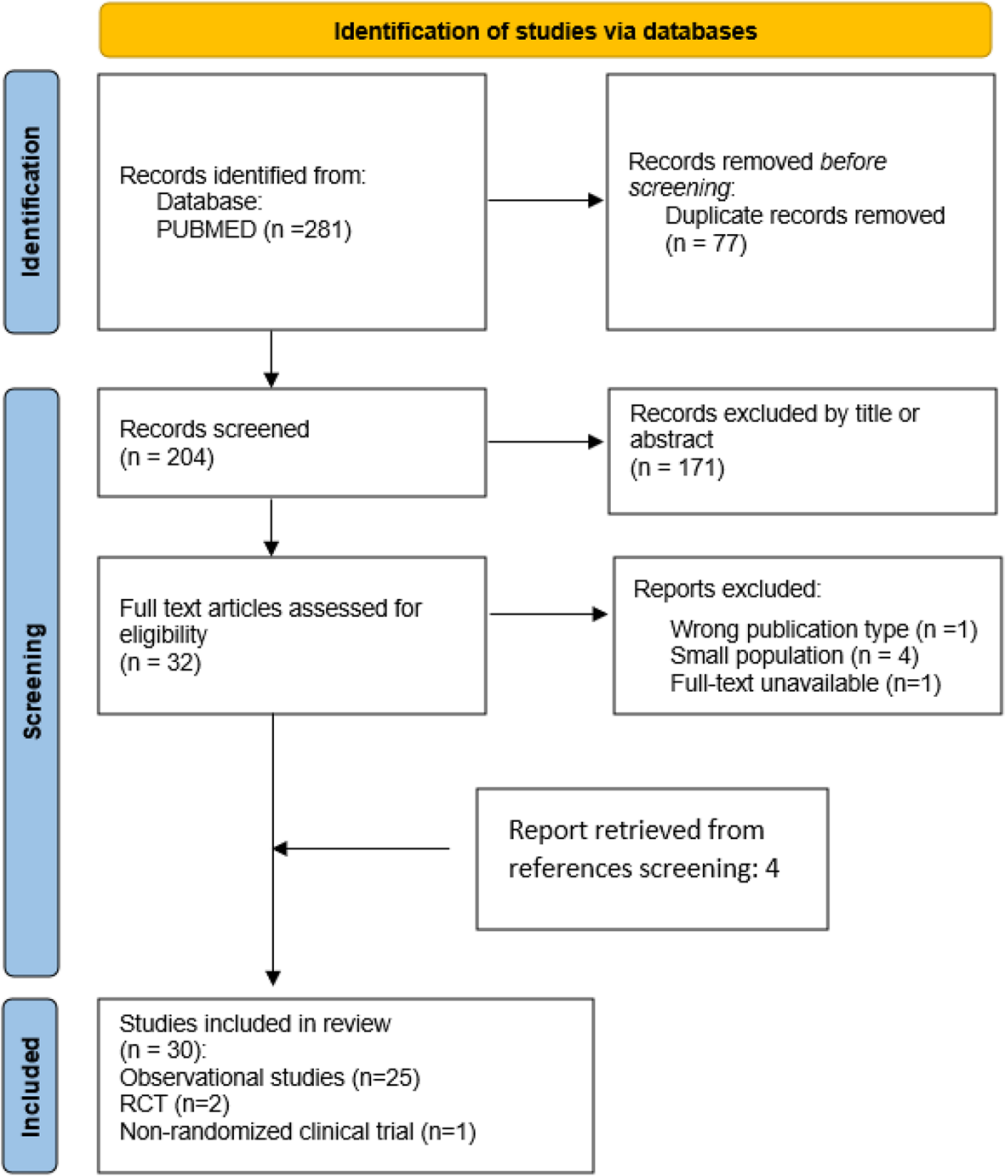
Flow diagram of literature search and data extraction

**Fig. 2:**
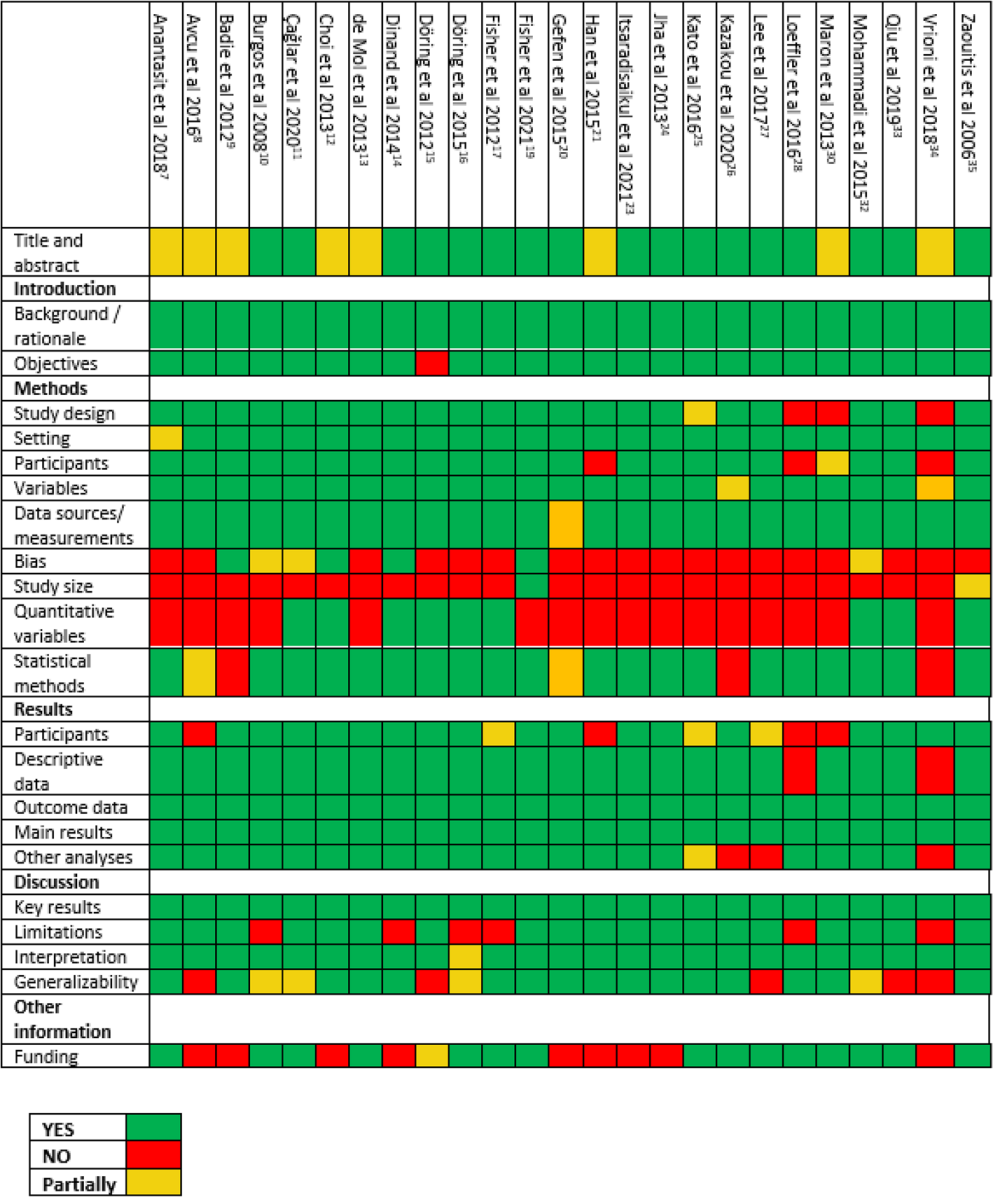
Adherence to STROBE recommendations

**Fig. 3:**
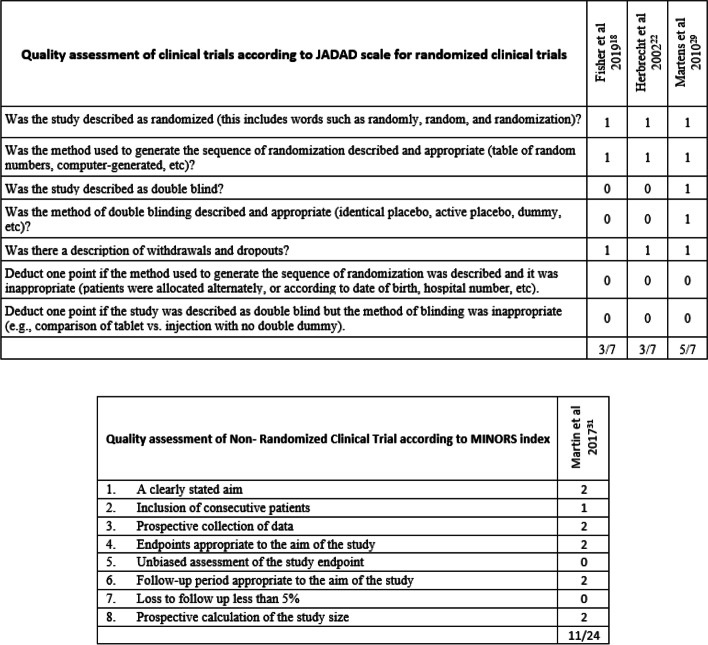
Clinical trials quality assessment

Five clinical trial (4 RCT and 1 non-randomized trial) and 25 observational studies were included in the review (Fig. 1). The quality assessment of selected studies is reported in Figs. 2 and 3. The characteristics and findings of selected studies are summarized in Table 1.

**Table 1:** Summary of findings

Author	Year	Country	Aim	Study design	Number of patients	Age	Results
Anantasit et al. [7]	2018	Thailand	To validate EORT/MSG 2008 definition in paediatric population Histology vs EORTC/MSG 2008	Retrospective Cross-sectional	256	1 m- 18 y	EORT/MSG 2008 sensitivity 100% specificity 36% PPV 33% NPV 100% EORT/MSG useful as screening tool Neutropenia described as risk factor for IPA
Avcu et al. [8]	2017	Turkey	To determine the utility of serum GM monitoring in early diagnosis of IA and its role in the management of children with ALL	Retrospective cohort study	141 3264 samples	Median age 55 m (range 3–208 m)	False positive: 52.1% Multiple consecutive positive tests increased the incidence of true-positive tests and introduction of antifungal therapy
Badiee et al. [9]	2012	Iran	To evaluate the diagnostic potential of EIA for GM, nested PCR and BDG test	Prospective matched cohort study	62 patients 230 samples 36 pts (129 samples) with IA suspicion 26 pts (101 samples) controls	Mean age 9.3 y	Galactomannan EIA: sensitivity 90%, specificity 92%, PPV 81.8% NPV 96%, likelihood ratios for positive results 11.25, for negative results 0.1 beta–D–glucan: sensitivity 50%, specificity 46%, PPV 26%, NPV 70.6%, likelihood ratios for positive results 0.9, for negative results 0.9 nested-PCR: sensitivity 80%, specificity 96.2%, PPV 88.9%, NPV 92.6%, likelihood ratios for positive results 21, and negative results 0.2 Galactomannan and nested-PCR tests are useful as non-invasive methods for diagnosis of IA in children. Beta–D–glucan test is not an efficient diagnostic tool in those with hematologic disorders
Burgos et al. [10]	2008	US	To describe risk factors, diagnostic tools, treatments and outcomes of IA in children	Retrospective cross-sectional study	139 pts	Median age 10.1 y (17d-18y)	*A. fumigatus* was the most often reported species Risk factors: immunosuppressive therapies and allogenic HSCT Most common site: lungs (59%) Most frequent radiologic finding: nodules 34.6% with 2.2% showing the crescent sign, 11% the halo sign and 43.1% cavitation Treatment: 45.8% received more than 3 concomitant antifungal agents, no superiority among antifungal agents was found
Caglar et al. [11]	2020	Turkey	To evaluate the diagnostic value of serum GM positivity for IA in children	Retrospective cohort study	70 patients 104 samples	Median age 5 y (1–16)	Consecutive GM positivity has higher PPVs independently from the cut-off value chosen
Choi et al. [12]	2013	Korea	To investigate the use of GM antigen assay as diagnostic tool in pediatric cancer and HCT patients; to assess the characteristics of patients with IA	Retrospective controlled cohort study	83 patients 23 IA group 60 non-IA group 640 samples	Median age IA 12.3 y (0.7–18.4) Non-IA 6.4y (0.3–18.7)	The false-positive rate was 18.3% Being younger than 3 years of age, having a solid tumor, and receiving HCT within 4 weeks from the test caused false-positive results (*p* < 0.05) The most common clinical site of IA was the lung (91.3%), and consolidation was the most frequent finding in chest CT scans (36.8%). The mortality at 12 weeks was 43.5% Having a positive GM assay at least twice is useful in diagnosing IA in pediatric patients with cancer and HCT recipients
de Mol et al. [13]	2013	Netherlands	To study the diagnostic value of BAL GM in immunocompromised children	Retrospective cross sectional study	47 pts 47 bronchoscopies	Median age 9.8 (1.1–18.2) y	BAL GM for proven and probable IPA: Sensitivity 82.4%, specificity 87.5%, PPV 82.4, NPV 87.5% A significant relation for BAL GM and abnormal chest CT (*p* = 0.01) BAL GM and serum GM correlated significantly BAL GM test had good diagnostic value in children with suspected IPA. The decision to continue or start antifungal therapy was mainly determined by the clinical suspicion of IPA based on chest CT-outcome, serum GM index values and failure of antibiotic therapy
Dinand et al. [14]	2016	India	To evaluate the use and optimal serum GM cut-off in children	Prospective cohort study	145 pts 211 febrile episodes	Median age 5 (0.5–19) y	Serum GA is sensitive to diagnose IA in pediatric patients with excellent NPV with a cut-off of 0.7. Two consecutive values of 0.7 increases specificity to 91.0%
Doring et al. [15]	2012	Germany	To analyse safety and efficacy of CAS and L-AmB in HSCT patients	Retrospective matched cohort study	60 pts received CAS 60 pts received L-AmB	Median age Cas group 9.5y L-AmB group 7.5y	Similar efficacy between prophylaxis with CAS and L-AmB after allogenic HSCT More drug-related side effects and an increased need for oral supplementation with potassium, sodium bicarbonate and calcium upon discharge in L-AmB receiving group
Doring et al. [16]	2015	Germany	To assess safety, feasibility, and efficacy of posaconazole compared to fluconazole and itraconazole in neutropenic children and adolescent	Retrospective matched cohort study	93 pts 31 fluconazole 32 itraconazole 30 posaconazole	Median age 12y (9 m-17.7y)	Posaconazole, fluconazole, and itraconazole are comparably effective in preventing invasive fungal infections in children Larger studies are required to define dose recommendations No statistical significant differences found in adverse events
Fisher et al. [17]	2012	US	To evaluate GM EIA as diagnostic tool in children after intensive chemotherapy or HSCT	Multicentre prospective cohort study	213 patients 1865 serum samples from 198 pts 886 urine samples from 183 pts 7 BAL samples from 4 pts	7.8 y	Serum GM testing specificity 95% Urine GM testing specificity 80% The urine test resulted in a higher false positivity rate, but it successfully identified the only case of probable IA Screening for GM, or a related antigen in urine, needs to be further evaluated as it may be useful in surveillance strategies
Fisher et al. [18]	2019	US and Canada	To compare the efficacy of CAS *vs.* fluconazole prophylaxis against proven or probable invasive fungal disease and IA in neutropenic patients following AML chemotherapy	Multicentre randomized open label clinical trial	257 CAS 260 fluconazole	Median age 9 (0–36) y	Prophylaxis with CAS compared with fluconazole resulted in significantly lower incidence of invasive fungal disease and proven and probable IA
Fisher et al. [19]	2021	US	To assess surveillance testing with GM EIA and BDG assay in children with AML receiving antifungal prophylaxis	Prospective cohort study	425 pts 209 fluconazole 216 CAS 6103 samples	Median age 10(0–25) y	NPV > 99% for GM EIA and BDG test alone and in combination Sensitivity and PPV 0% GM EIA and BDG test should be discouraged for surveillance in patients with AML receiving antifungal prophylaxis
Gefen et al. [20]	2015	Israel	To investigate serial serum GM assay screening on IPA diagnosis in children with HSCT or high risk leukemia	Prospective cohort study	34 pts 510 samples in neutropenic children	Median age 8.5 y (6 m-19y)	GM assay: sensitivity 0.8, specificity 0.66, PPV 0.22 and NPV 0.96
Han et al. [21]	2015	Korea	To characterize IPA in children with hematological/oncological disorders	Retrospective matched cohort study	166 pts		Neutropenia lasting more than 2 weeks (51.4% vs. 21.9%, *p* < 0.001) and halo signs at chest CT (78.4% *vs*. 40.7%, *p* < 0.001) were more frequent among children with IPA Early use of chest CT in children at risk of prolonged neutropenia could be helpful for early IPA diagnosis
Herbrecht et al. [22]	2002	Multicentre Invasive Fungal Infection Group of EORTC	To compare voriconazole with AmB as primary therapy of IPA	Randomized, unblinded clinical trial	144 pts voriconazole 133 AmB	Mean age voriconazole group: 48.5 (13–79)y AmB group: 50.5 (12–75) y	Voriconazole led to better responses, improving survival with less severe side effects than amphotericin B
Itsaradisaikul et al. [23]	2021	Thailand	To evaluate 1-year incidence of IFD after itraconazole prophylaxis in HSCT children; to identify risk factors, etiology and adverse events	Retrospective cohort study	170 pts	Median age 8.43 (5.41–12.36) y	Itraconazole did not showed a excellent efficacy in preventing IFD after HSCT. It could be used in resource-limited settings. It requires appropriate drug level monitoring if used
Jha et al. [24]	2013	India	To evaluate the role of GM assay in IA diagnosis in children on treatment for hematological malignancies and to identify the best cut-off values	Prospective cohort study	78 pts 100 ferbile episodes	Mean age 6.1 y (1.5–13)	Best results with cut-off value of 1.0 GM assay (cut-off value 1.0): Sensitivity 60%, specificity 93%, PPV 75, NPV 87 A higher value of GM related with pulmonary nodules (*p* = 0.037) and mortality (*p* = 0.001)
Kato et al. [25]	2016	Japan	To identify the daily therapeutic dose in children; to analyze association between voriconazole concentration and clinical outcomes	Retrospective cohort study	20 pts 111 samples	Median age 9.5 (0–17) y	younger age and oral administration were associated with lower plasma voriconazole concentrations (*p* < 0.01). Unfavourable outcome was associated with low concentrations of voriconazole (*p* = 0.01) Higher doses are required in younger children and in case of oral administration
Kazakou et al. [26]	2020	Greece	To evaluate the incidence of IFD in children with hematological malignancies and determine the clinical characteristics, risk factors, diagnosis, treatment efficacy and outcome	Retrospective cohort study	297 pts	Mean age 6.64 (2–13) y	Most common underlying disease: ALL (79%) Most common site of infection: lungs (66.7%) Identified species: *Aspergillus* spp. (58.3%) Most prescribed treatment: L-AmB The crude mortality rate was 33.3%
Lee et al. [27]	2017	Korea	To determine safety and efficacy of the combination of Voriconazole and CAS to treat IFDs	Retrospective cross-sectional study	22 pts	Mean age 5 3 (0.8–13.3) y	Voriconazole plus CAS is an effective and safe treatment for serious IFD in children with leukemia
Loeffler et al. [28]	2017	Germany	To determine the use of GM assay combined with PCR assay in HSCT recipients	Retrospective cohort study	39 pts 543 samples	Median age male: 9.5 (4–21) y female: 10 (3–19) y	GM assay: specificity 89%, sensitivity 67%, PPV 50% NPV 100% PCR assay: specificity 63%%, sensitivity 100%, PPV 27% NPV 100% combined monitoring for GM and fungal DNA results in a higher diagnostic accuracy
Maertens et al. [29]	2010	US and Europe	To evaluate CAS *vs* L-AmB for Empiric antifungal therapy in children with persistent fever and neutropenia	Randomized double blind clinical trial	83 pts 57 CAS 26 L-AmB	Range 2–17 y	CAS and L-AmB were comparable in tolerability, safety and efficacy as empiric antimicotic therapy
Maron et al. [30]	2013	US	To compare etiology, predisposing factors and outcomes of IFD in AML patients before and after implementation of voriconazole prophylaxis	Retrospective cohort study	19 pts AML97 (no fungal prophylaxis) = 12 pts AML02 (voriconazole prophylaxis) = 11 pts	Median age AML97: 11 (0.3–21) y AML02: 8(1–19) y	Voriconazole prophylaxis was associated with improved survival and a significant reduction in aspergillosis
Martin et al. [31]	2017	Multicentre Asia, Europe and North America	To evaluate safety, efficacy and exposure–response of voriconazole as treatment for IA, IC and EC	Prospective open-label, non-comparative phase 3 study	53 pts 31 IA 22 IC/EC	Mean age IA 11.9 (SD 3.5) IC/EC 9.5 (SD 4.5)	In IA cohort: 22.6% treatment related hepatic AE and 16.1% visual AE; all-causality mortality 14.3% at week 6, no deaths attributed to voriconazole Voriconazole is effective in patients with IA with a favourable risk–benefit balance
Mohammadi et al. [32]	2015	Iran	To evaluate the efficacy of BAL GM in immunocompromised patients	prospective case–control study	16 pts immunocompromised, with possible/proven IPA by EORT/MSG criteria 54 controls	Mean age (IPA pts): 8.4 (11–15) y	BAL GM using an OD index of ≥ 0.5: Sensitivity 87.5% PPV 93.33% High diagnostic value of BAL GM in immunocompromised children with IPA
Qiu et al. [33]	2019	China	To evaluate the diagnostic value for IPA of serum GM combined with CT in children after HSCT	Retrospective case control study	46 cases 95 controls	Mean age Cases 7 ± 3.7 y Controls 6.2 ± 3.5y	GM testing combined with CT evaluation: PPV of 0.764, and NPV of 0.872, Sensitivity 0.793, and specificity 0.852 The combination of serum GM and chest CT might be used for early diagnosis of IPA in HSCT patients
Vrioni et al. [34]	2018	Greece	To define the use of GM serum assay and PCR as routine methods for IA in immunosuppressed children	Prospective cohort study	156 pts 744 samples	Age range 5 m-14y	Agreement of the two methods: 90% of pts, 96.1% of samples The combination of GM and PCR had a high diagnostic accuracy in consecutive samples (twice weekly)
Zaoutis et al. [35]	2006	US	To describe the incidence and outcomes of IA in children	Retrospective cohort study	666 pts	Median age 13 (IQR 8–15)	Highest incidence of IA in children with HSCT (4.5%) and AML (4%). The overall in- mortality of immunocompromised children was 18%. Children with malignancy and IA were at higher risk for death Children with IA had a longer hospital stay and higher hospital charges

*Abbreviations*: *ALL* Acute Lymphoblastic Leukemia, *AML* Acute Myeloid Leukemia, *AML97 and AML02*: multicentre protocols for pediatric patients with AML, *BAL* Bronchoalveolar lavage, *BDG* β-D-glucan, *CAS* Caspofungin, *CT* Computed tomography, *d* days, *EIA* Ezyme Immunoassay, *EC* Esophageal candidiasis, *EORTC/MSG* European Organization for Research and Treatment of Cancer/Invasive Fungal Infections Cooperative Group and The National Institute of Allergy and Infectious Disease Mycoses Study Group, *GM* galactomannan, *HSCT* Hematopoietic Stem Cell Transplant, *IA* Invasive Aspergillosis, *IC* Invasive candidiasis, *IFD* Invasive Fungal Disease, *IQR* Interquartile range, *L-AmB* Liposomal amphotericin B, *m* months, *NPV* Negative predictive value, *OD* Optical density, *PCR* Polymerase Chain Reaction, *PPV* Predictive positive value, *Pts* Patients, *y* years, *US* United States

### What are the main risk factors for IPA in children?

In immune-competent hosts, occasional colonisation of fungal conidia is controlled by the immune system. In contrast, in immune-compromised subjects, an invasive infection can occur, primarily affecting the lungs. However, dissemination to the central nervous system is reported in up to 30% of cases. The IPA development depends on the patient's immune status and lung parenchyma characteristics (Table 2).

**Table 2:** Host factors for invasive pulmonary aspergillosis

Host factors for invasive pulmonary aspergillosis
1) Prolonged neutropenia (< 500 cells/mm^3^ for > 10 days)
2) Transplantation (higher risk for lung and hematopoietic stem cell transplantation)
3) Prolonged (> 3 weeks) high-dose corticosteroid therapy in the past 60 days
4) Treatment with other T-cell immunosuppressants
5) Treatment with B-cell immunosuppressants
6) Hematological malignancy (higher risk for leukemia)
7) Severe primary immunodeficiencies (e.g., CGD, WAS)
8) Acute grade III-IV GVHD with gut, lung or liver involvement and steroid-resistant

*Abbreviations*: *CGD* Chronic granulomatous disease, *WAS* Wiskott-Aldrich syndrome, *GVHD* Graft-versus-host disease

IPA represents an emerging problem and is one of the leading causes of morbidity and mortality in immune-compromised patients. The incidence of IPA in children receiving chemotherapy is high. It is associated with increased morbidity and death, with highest rates in patients with acute myeloid leukaemia (AML), recurrent leukaemia, and those undergoing hematopoietic stem cell transplantation (HSCT) [10, 21, 26]. A large multicenter study conducted by Zaoutis et al. in 2006 including 666 children with invasive aspergillosis found that 60–75% of cases are oncologic patients with mortality rates as high as 85% [35]. The risk was higher in case of allogeneic transplantation than in autologous transplantation and in cases of severe graft versus host disease (GVHD). One of the larger cohorts of patients with IPA was described by Burgos et al. in a multicenter retrospective analysis in 2008; they examined 139 paediatric patients with invasive aspergillosis, 80% of which had lung involvement [10]. The most common underlying conditions were haematological malignancies (87/139, 62.6%), followed by inherited immunodeficiencies (16/139, 11.5%), solid organ transplant (16/139, 11.5%), solid tumour (9/139, 6.5%). In particular, 51 patients (26.6%) underwent allogenic HSCT, which was identified as the most critical risk factor for overall mortality in invasive aspergillosis (OR 6.14 – 95% CI 2.67–16.21). Severe neutropenia, defined as neutrophil count below 500 cells/mm^3^, is the main single risk factor for the development of IPA; in the abovementioned study, it accounted for about 59% of aspergillosis cases. In addition, most hemato-oncology patients presented additional immunosuppression due to prolonged therapy with high-dose steroids or immunosuppressive drugs such as cyclosporine or tacrolimus [10, 26].

Considering primary immunodeficiencies, invasive aspergillosis may be the presenting manifestation or a frequent complication in the first two decades of life in children with chronic granulomatous disease (CDG), a condition caused by neutrophil dysfunction. In such patients, *Aspergillus* spp. can cause brain abscesses, osteomyelitis and lung damage. Invasive aspergillosis may also occur in children with Wiskott-Aldrich syndrome (WAS), in which neutrophil chemotaxis and lymphocyte function is impaired. Burgos et al. found that CDG accounted for almost 50% of invasive aspergillosis cases among children with primary immunodeficiency (7/16). In contrast WAS was found to be the predominant underlying condition (81/122, 66%) in the larger study by Zaoutis et al., followed by CDG (21/122, 17%) [10, 35]. Among all children with invasive aspergillosis, the highest incidence was observed in those with WAS (30%), followed by CGD (6.5%), allogenic HSCT (4.5%) and AML (3.7%) [35]. Nevertheless, immunodeficiency is not the only risk factor for IPA, since patients hospitalised in intensive care units, children with chronic obstructive pulmonary disease, emphysema, or those affected by chronic diseases such as CF, are also at increased risk. Nevertheless, Zaoutis et al. observed that only 5 out of over 11,000 patients with CF were diagnosed with IPA [35].

### What are the main diagnostic tools for IPA in children?

The diagnosis of IPA still represents a challenge for the clinician today. Despite the introduction of new diagnostic techniques, the high mortality rate of this condition is mainly due to diagnostic delay [12]. An early diagnosis and prompt therapy is crucial for a better outcome, especially in the immune-compromised child. Maintaining a high index of suspicion in patients with multiple risk factors is essential. An in-depth diagnostic investigation is necessary in case of fever unresponsive to antibiotic therapy, or cough with sputum and dyspnoea in high-risk children. Chest pain with pleural involvement (due to small pulmonary infarcts) and haemoptysis may be associated with the clinical picture of IPA. When infection disseminates to the central nervous system, seizures or radiological alterations consistent with cerebral infarcts, intracranial haemorrhages, or epidural abscesses may occur.

No specific biochemical and/or instrumental tests allow a diagnosis of certainty. Therefore, diagnostic tests should be performed sequentially starting with the least invasive ones. The result of each test should be considered according to the clinical and immune status of the patient. Currently validated diagnostic criteria for adults, which are also used in some paediatric studies [12, 13] distinguish between “proven”, “probable” and “possible” IPA. In a recent Consensus of the European Organization for Research and Treatment of Cancer and the Mycoses Study Group (EORTC/MSG), the definition of invasive fungal disease has been revised, with no change from the previous 2008 classification (Table 3) [36]. These criteria have shown to have an excellent sensitivity but low specificity in detecting possible or probable cases of IPA [7]. The least invasive test for identification of aspergillus infection is the blood assay of galactomannan antigen (GM), a wall component released in the blood due to its growth. GM can also be assayed in other biological fluids, such as bronchoalveolar lavage fluid (BAL) or cerebrospinal fluid, in rare cases of neurological involvement, being more reliable than the blood value [12, 13]. The sensitivity and specificity of the blood assay depend on various components, such as the underlying pathology, current therapies or cut-off used by the laboratory, since there is currently no consensus about negative values. Choi et al. analysed 749 blood samples from 99 oncological children and showed that blood assay of GM was more reliable in cancer patients in detecting invasive aspergillosis (sensitivity: 91.3%; specificity: 81.7%; false positives: 18%) [12]. Similar results were found in the studies of Badiee et al. and Fisher et al. on 62 and 198 paediatric haemato-oncology patients, respectively [9, 17]. Recent studies showed variable sensitivity, specificity and positive predictive value (PPV) of single serum GM determination. On the other hand, consecutive positive tests had higher PPV, especially in a compatible clinical and radiological context [8, 11, 20, 24]. This variability is, to some extent, linked to that of the cut-off used to define GM positivity. In the prospective study of Dinand et al. on 145 neutropenic patients, the optimal cut-off value for single GM determination was found to be 0.7 with sensitivity and specificity both around 82% and negative predictive value (NPV) of 94%, and specificity increased to 91% in case of positivity on a consecutive test [14]. In a recent cross-sectional study of Çağlar et al. on 70 patients with haematological malignancies, consecutive GM positivity displayed higher PPVs independently from the cut-off value chosen [11]. Considering GM on BAL, Mohammadi et al. reported sensitivity and positive predictive values of 87.5% and 93.33% respectively, using a cut-off of ≥ 0.5. Moreover in 7 out of 16 cases of IPA, serum GM was negative, while their BAL GM was positive [32].

**Table 3:** Diagnostic criteria for invasive aspergillosis [10]

Diagnosis	Criteria
Certain	Histological or cytological evaluation of lung tissue with hyphae on needle aspiration or biopsy in which hyphae or melanized yeast-like forms are associated to tissue damage *or* Positive culture test for Aspergillus on pulmonary specimen taken by sterile procedure *and* Clinical or radiologic abnormalities consistent with infection
Probable	At least 1 host factor (tab. 2) *and* Mycological evidence - positive microscopy or culture for *Aspergillus* on sputum, BAL bronchial brush, or aspirate - positive *Aspergillus* PCR (at least 2 tests) - positive antigenic assay^a^ *AND* Clinical criteria consistent with infection^b^
Possible	At least 1 host factor (tab.2) *and* Clinical criteria compatible with infection^b^

^a^Positive antigenic assay: detection of galactomannan in plasma, serum, BAL, or CSF. β-D-glucan was not considered to provide mycological evidence of any invasive mold disease

^b^Clinical criteria compatible with infection: characteristic infiltrates on CT (dense, well-circumscribed lesions with or without halo sign, air crescent sign, or cavity), tracheobronchitis diagnosed by bronchoscopy, or infiltrates that are uncharacteristic but associated with specific pulmonary symptoms or signs (e.g., pleural pain, haemoptysis)

*Abbreviations*: *BAL* Bronchoalveolar lavage, *CSF* Cerebrospinal fluid, *CT* Computed tomography, *PCR* polymerase chain reaction

Another *Aspergillus* antigen is β-D-glucan, which is shared with other fungal species such as *Candida* spp. and *Pneumocystis* spp. Data available in children are scarce, and an optimal cut-off is unknown, as mean β-D-glucan levels are higher in immunocompetent children than in adults. The combined assay of the two wall components may the reliability of the tests [9] even if, to date, β-D-glucan is not recommended for screening or evaluating suspected IPA in high-risk patients. At last, neither GM nor β-D-glucan (alone or in combination) can be used as a screening marker in neutropenic patients undergoing antifungal prophylaxis, since none of them has shown an acceptable sensitivity [19].

Evidence regarding the use of other biochemical parameters, such as total IgE, *Aspergillus*-specific IgE or peripheral eosinophil counts as significant support for the diagnosis of IPA is lacking.

Radiological examinations are essential for the diagnosis of IPA. Nevertheless, recent studies showed that the current radiological criteria used in adults are not applicable in children [10, 12]: radiographic findings considered typical of IPA in adults are not seen in the majority of children with IPA, and unspecific findings are detected more often in immunocompromised children. I In case of multiple nonspecific nodules t chest-xrays (up to 35% of cases) [10], a differential diagnosis with viral (from Cytomegalovirus or Adenovirus) or bacterial (*Nocardia*) pneumonia is required. Similarly, high-resolution chest computed tomography (HRCT), the most useful imaging tool, rarely evidences pathognomonic lesions of IPA in children, such as “halo sign” (area of ground-glass opacity surrounding a nodule), “air crescent sign” (area of increasing radiolucency in a region of nodular opacity), or cavitary lesions [10, 12]. A single-centre case–control study including 141 children with neutropenic fever lasting more than 96 h showed that the combination use of chest CT scan and serum GM testing was useful for early diagnosis, with PPV and NPV of 76% and 87%, respectively [33]. In the multicenter analysis of Burgos et al., including 110 children with IPA, 61 of them had 2 or more radiological findings on chest CT or plain radiograph, with pulmonary nodules being the most common; (65/110, 59%); on the other hand, halo sign and air crescent were found in smaller percentages of patients (10.9% and 2.2% respectively) [10]. Thus, radiological findings are often unsatisfactory and further examination is required for diagnostic confirmation. Molecular testing through Polymerase Chain Reaction (PCR) on blood or BAL has recently been included among diagnostic criteria for probable aspergillosis. However, its role in patient management has not been established yet. Badiee et al. reported a high NPV of *Aspergillus*-specific PCR as a screening tool [9], in line with two other studies showing that a combination of GM and PCR testing could be used for screening or diagnostic purposes, especially when tested on consecutive samples [28, 34], However, despite promising results, *Aspergillus* PCR is not recommended for routine use yet, since its standardization and validation are lacking.

The current gold standard for the diagnosis of IPA is lung biopsy. It is an invasive exam difficult to perform in clinically compromised patients, considering the frequent association between thrombocytopenia and advanced forms of IPA. Therefore, lung biopsy should be performed only when less invasive procedures have not been conclusive. In most cases, lung biopsy is performed through a CT-guided transbronchial route with BAL collection. Since the collected sample is often quantitatively insufficient and the sensitivity of culture examinations is poor, PCR techniques have been introduced to identify *Aspergillus* DNA on histological samples [9].

Lastly, hyphae in lung tissue or a positive culture for *Aspergillus* spp. on the same site or on normally sterile biological fluids allows the diagnosis of IPA in patients with a suggestive clinical or radiological picture. In high-risk children with a clinical and radiological suspect of IPA, GM antigen positivity on serum or BAL or fungal growth in BAL are required for IPA diagnosis and in these cases lung biopsy is unnecessary.

### What is the first-line agent for IPA in children?

The mortality rate of IPA in untreated paediatric patients is close to 100% and remains very high even with aggressive drug therapy. For this reason, empirically based antifungal therapy must be started as soon as possible in children at high risk of developing invasive forms of aspergillosis or otherwise undergoing diagnostic evaluation [7, 27]. In paediatric age, the major difficulties in the therapeutic choice are related to the lack of RCTs on large sample sizes and the paucity of unambiguous data regarding the first-choice drug class, the duration of therapy, and the outcomes to evaluate the clinical response. Most of the available data refer to studies carried out on adults. To date, the first-choice treatment options in children older than 2 years of age are voriconazole, and liposomal amphotericin B. Voriconazole is not approved in children younger than 2 years of age, and the optimal dose is unclear, hence liposomal amphotericin B is the drug of choice. Nevertheless, limited safety data for the use of liposomal amphotericin B in neonates are available. Safety and efficacy of voriconazole were evaluated on 31 paediatric patients treated for 6–9 weeks for invasive aspergillosis in a prospective study showing that voriconazole is generally effective in paediatric patients, with a favourable risk–benefit balance and an overall safety profile similar to adults [31]. However, there is no firm data on the dosing of voriconazole in children aged 2 to 12 years.

In a large multicenter randomized trial on 277 patients older than 12 years old comparing children treated with voriconazole to the ones treated with amphotericin B, the first group was found to have a higher response rate, better survival at 12 weeks of treatment and fewer adverse events [22]. Nevertheless, there is no clear evidence to prefer one of the two drugs, since there is a lack of controlled and randomized comparison studies. On the other hand, there is an unequivocal need to monitor voriconazole plasma levels during therapy, especially in younger patients requiring higher doses [25]; plasma concentrations of 1—5 mg/l are usually considered adequate for prophylaxis and treatment of IPA. The dosages of most common antifungal drugs used for paediatric invasive aspergillosis are summarized in Table 4. Lastly, a few studies have investigated the safety and efficacy of drug combination in children; in a small retrospective study in leukemic patients with invasive fungal disease, 9 of which with invasive aspergillosis, the combination of voriconazole and caspofungin was safe and effective but further studies are needed [27]. Data providing evidence for biologic drug use in ABPA are scarce at the moment [37].

**Table 4:** Dosages of most common antifungal drugs in paediatric IPA

Drug	Dosage by age group				Adverse events
	**Newborns**	**1–24 months**	**2–12 years**	**13–18 years**	
Voriconazole e.v (mg/Kg/die)	Not approved	Not approved	14 (in 2 doses)	8 (in 2 doses)	Visual disturbances Hepatotoxicity Hypersensitivity Skin rash
Voriconazole per os (mg/die)	Not approved	Not approved	400 (in 2 doses)	400 (in 2 doses)	
Conventional amphotericin e.v (mg/Kg/die)	1–1,5	1–1,5	1–1,5	1–1,5	Nephrotoxicity Electrolyte disturbances Hypersensitivity
Amphotericin lipid formulation (mg/Kg/die)	5 3–5 NA	5 3–5 3–4	5 3–5 3–4	5 3–5 3–4	Mild nephrotoxicity Electrolyte disturbances
Caspofungin (mg/m^2^/die)	25	50 (max 70); 70 per day 1	50 (max 70); 70 per day 1	50 (max 70); 70 per day 1	Fever Hepatotoxicity Cutaneous rash Tachycardia Headache
Posaconazole per os (mg/die)	NA	NA	NA	800 (in 2 o 4 doses)	Hepatotoxicity Nausea and vomiting Headache
Itraconazole per os (mg/Kg/die)	NA	NA	5 (in 2 doses)	5 (in 2 doses)	Abdominal pain Nausea Skin rash

*Abbreviations*: *NA* Not available, *BLC* Amphotericin B lipid complex, *L-AMB* Amphotericin B liposomal, *ABCD* Amphotericin B colloidal dispersion

### What is the optimal duration of treatment for IPA in children?

The duration of antifungal therapy has not been established and often needs to be individualized according to the child immune status. In a paediatric population examined in 2007, the mean duration of therapy was 93 days (range 1–880) with a partial response in 45% of cases. However, cases of severely immune-compromised patients in whom therapy was continued indefinitely have also been described [38]. In the prospective study of Martin et al., patients were treated for 6–9 weeks according to clinical response [31]. The most relevant issues in paediatric patients are the sequence of drugs to be used and the clinical parameters to be followed to evaluate the efficacy of therapy. Two main RCTs have been performed in the past in paediatric age groups [29, 39]. Although the expected outcomes are different, they agree that clinical improvement and fever relief for at least three consecutive days are the best evidence of therapy success. Even if regular dosing of GM antigen, after a first positive determination, could be helpful to guide antifungal therapy duration, a negative finding is not sufficient to discontinue therapy [13].

### What are the main prophylactic drugs used for IPA in children?

Most of the evidence available in adults suggests the use of posaconazole for prophylactic purpose. It can be used in patients aged older than 13 years, neutropenic, with de novo or recurrent forms of AML, recurrent forms of acute lymphoblastic leukemia, stem cell transplant recipients or affected by GVHD [16] oral voriconazole or itraconazole are the drugs of choice for children aged 2 to 12 years [16, 23, 30]. In patients younger than 2 years of age or unable to take oral drugs, liposomal amphotericin B may be used [15], or, in those older than 2 years, intravenous voriconazole may be prescribed [39]. In a multicenter RCT including 517 patients from 3 months to 30 years of age, caspofungin was found to be superior to fluconazole in reducing invasive fungal diseases, including invasive aspergillosis [18].

## Conclusions

Invasive pulmonary aspergillosis is a life-threatening condition and one of the leading causes of morbidity and mortality in fragile patients, however its diagnosis and management continue to be a clinical challenge. Among children, haematological malignancies, a previous organ transplant, and other primary or acquired immunodeficiency are the main risk factors for IPA**.**

GM antigen detection is a first-line diagnostic tool for high-risk patients with suspected aspergillosis and consecutive tests on BAL provide a high PPV, especially in a compatible clinical and radiological picture. No solid data regarding β-D-glucan diagnostic role in children are available, and PCR assays are not standardized nor validated for *Aspergillus* spp., therefore those tests should not be routinely used.

Voriconazole is currently the drug of choice in children older than 2 years. Liposomal amphotericin B should be administered in children younger than 2 years old or if voriconazole is contraindicated,. An unequivocal recommendation on the duration of treatment is missing since literature data are inconsistent. Clinical status and inflammatory and microbiological findings can guide an individualized therapy length. Voriconazole or itraconazole is suggested in children younger than 13 years old needing antifungal prophylaxis due to the paucity of safety data regarding posaconazole, while it can be safely used in older children.

Data about the diagnosis and management of IPA in children and adolescents are scarce, and no significant progress has been made in the last decades, suggesting that further high-quality studies are needed to improve clinical strategies.

## Data Availability

Not applicable.

## Abbreviations

IPA: Invasive pulmonary aspergillosis
PCR: Polymerase chain reaction
RCT: Randomized controlled trials
CF: Cystic fibrosis
PRISMA: Preferred reporting items for systematic reviews and metanalyses
AML: Acute myeloid leukaemia
HSCT: Hematopoietic stem cells transplantation
GVHD: Graft versus host disease
OR: Odds ratio
CI: Confidence interval
CGD: Chronic granulomatous disease
GM: Galactomannan antigen
BAL: Bronchoalveolar lavage
PPV: Positive predictive value
NPV: Negative predictive value
CT: Computed tomography
DNA: Deoxyribonucleic acid
